# Pollen collection by the western honeybee and common eastern bumble bee foraging in a common landscape and applications for agri-environment schemes

**DOI:** 10.1098/rsos.240675

**Published:** 2025-03-05

**Authors:** Danny Minahan, Johanne Brunet

**Affiliations:** ^1^ B. Triwaks Bee Research Center, Department of Entomology, The Robert H. Smith Faculty of Agriculture, Food and Environment, The Hebrew University of Jerusalem, Rehovot, Israel; ^2^ Department of Integrative Biology, University of Wisconsin, Madison, WI, USA; ^3^ Brunet Research, Madison, WI, USA; ^4^ Vegetable Crops Research Unit, USDA–Agricultural Research Service, Madison, WI, USA

**Keywords:** bumble bee, floral choice, foraging strategy, honeybee, pollen diversity, resource availability

## Abstract

Agricultural landscapes often provide an impoverished environment for bees given their limited plant and pollen diversity. Agri-environment schemes (AES) such as flower strips have been developed to improve the quality of the agricultural environment for bees but their efficacy varies with their composition and, for specific pollinators, with the value of the available plant species. This study provides a detailed report of the pollen collection patterns of two bee species, the western honeybee (*Apis mellifera* L.) and the common eastern bumble bee (*Bombus impatiens* Cresson), over their foraging season. We compared the floral constancy, pollen richness and diversity of the two bee species, and the pollen morphotypes of bee-collected pollen in relation to resource availability. The honeybee was more flower constant while the bumble bee collected a greater family level diversity of pollen. While both bee species collected similar resources over their entire foraging season, the preferred morphotypes in given surveys differed between bee species. Neither bee species collected resources based on their availability but indicated patterns of preference and avoidance. We discuss how such knowledge can inform the composition of AES to best sustain these pollinators in more impoverished depauperate agricultural landscapes.

## 1. Introduction

Bees are important pollinators of a variety of crops and wildflowers, with their ecosystem services being valued at €153 billion [1]. Honeybees have been introduced for crop pollination around the globe, and when present, contribute to approximately 19% of all crop plant visits in a region [2]. Wild bees also contribute to increases in fruit production for pollinator-dependent crops [3]. Bees collect both pollen and nectar from plants, and pollen provides the primary source of macronutrients such as protein, lipids, sterols and minerals, which are important for the growth and development of bee larvae and colonies [4–7]. However, not all plant pollen is equivalent in the relative amount of nutrients or their ratios [8,9]. Social bees will gather pollen from a variety of plant taxa to maintain a nutritional balance [10,11] that ensures appropriate growth and development [12], behavioural performance [13] and to mitigate other stressors such as infection from parasites [5].

Agricultural landscapes often provide an impoverished environment for bees given their limited plant and pollen diversity [14,15]. Thus, collecting pollen from a variety of plant taxa can become a challenge in high intensity agricultural landscapes, where the limited resource availability often leads to a reduction in bee abundance and diversity with negative consequences for ecosystem services [16,17]. But agricultural landscapes can also be a challenge for managed pollinators like honeybees, because their nutritional requirements may not be met in a depauperate agricultural environment [18]. To improve the quality of the agricultural environment, agri-environment schemes (AES) have been developed and some of these practices include planting flower-rich field margins, hedgerows, flower strips and plots along crop field edges or in the vicinity of agricultural fields [16,17,19–21]. These multifunctional areas provide diverse resources for pollinators [22], and their presence has been shown to increase pollinator abundance and diversity in the margins themselves [23].

The success of AES such as flower strips is affected by a variety of factors such as the age of the strip, the surrounding landscape, the composition of the flower strip and the quality of the control relative to the strip itself [17,19,24]. For example, wildflower strips are often more effective at increasing insect diversity relative to strips with only cultivars or a mixture of cultivars and wildflowers [20], and native seed mixtures with a diversity of plant species are particularly effective [25]. The efficacy of flower strips and other AESs is affected by its composition [20,25] and, for specific pollinators, depends on the value of the available plant species [26,27]. Moreover, the success of agri- environment schemes varies with their intended goal. While wide success has been observed in studies aiming at increasing insect diversity and richness [17], no consistent pattern has been detected with respect to pollinators spilling over into the crop fields and positively influencing crop yield [28]. In fact, the results of two meta-analyses found no overall increase in crop yield following the implementation of flower strips [21,23].

Different groups of bees differ in how they use semi-natural areas relative to common mass flowering crops with implications for AES management strategies. While honeybees tend to forage on mass-flowering crops, wild bees prefer semi-natural habitats and bumble bees are more habitat generalists [29]. The information about resource location and quality transmitted via waggle dances by honeybees may affect their foraging at the more abundant monofloral resource patches, while the more exploratory bumble bees would explain them dispersing more evenly among all available resources. The waggle dance can also shorten the foraging distance traveled by honeybees and reduce their average colony foraging area [30]. Based on these differences in foraging behaviours between the two bee species, we further predict bumble bees would be less flower constant and bring richer and more diverse pollen back to their colonies. We also predict honeybees would preferentially visit the more abundant pollen morphotypes throughout their foraging season but cannot predict which pollen morphotypes bumble bees may prefer. We expect these two bee species to visit different plants throughout their foraging season in order to optimize pollen collection.

Here, we provide a detailed report on the pollen collection patterns of two bee species, the western honeybee (*Apis mellifera* L.) and the common eastern bumble bee (*Bombus impatiens* Cresson), in one agricultural landscape over their foraging season. We test the predictions concerning flower constancy, pollen richness and pollen diversity collected by these two bee species. By combining the information about morphotypes collected by the bees to the availability of morphotypes in the resources, we gain a better understanding of how plant abundance affects bee foraging patterns and highlight preference patterns of bees for less abundant plants. In doing so, we discuss some issues inherent to evaluating resource availability and comparing bee-collected pollen to resource abundance and provide potential solutions. Based on information gained from this study, combined with previous studies, we highlight plant groups beneficial to these two bee species. We discuss how knowledge of pollen foraging patterns of bee species can inform the composition of flower strips and other AESs to best sustain these pollinators in more impoverished agricultural landscapes.

## 2. Material and methods

### 2.1. Study area and bee species

**Figure 1.**
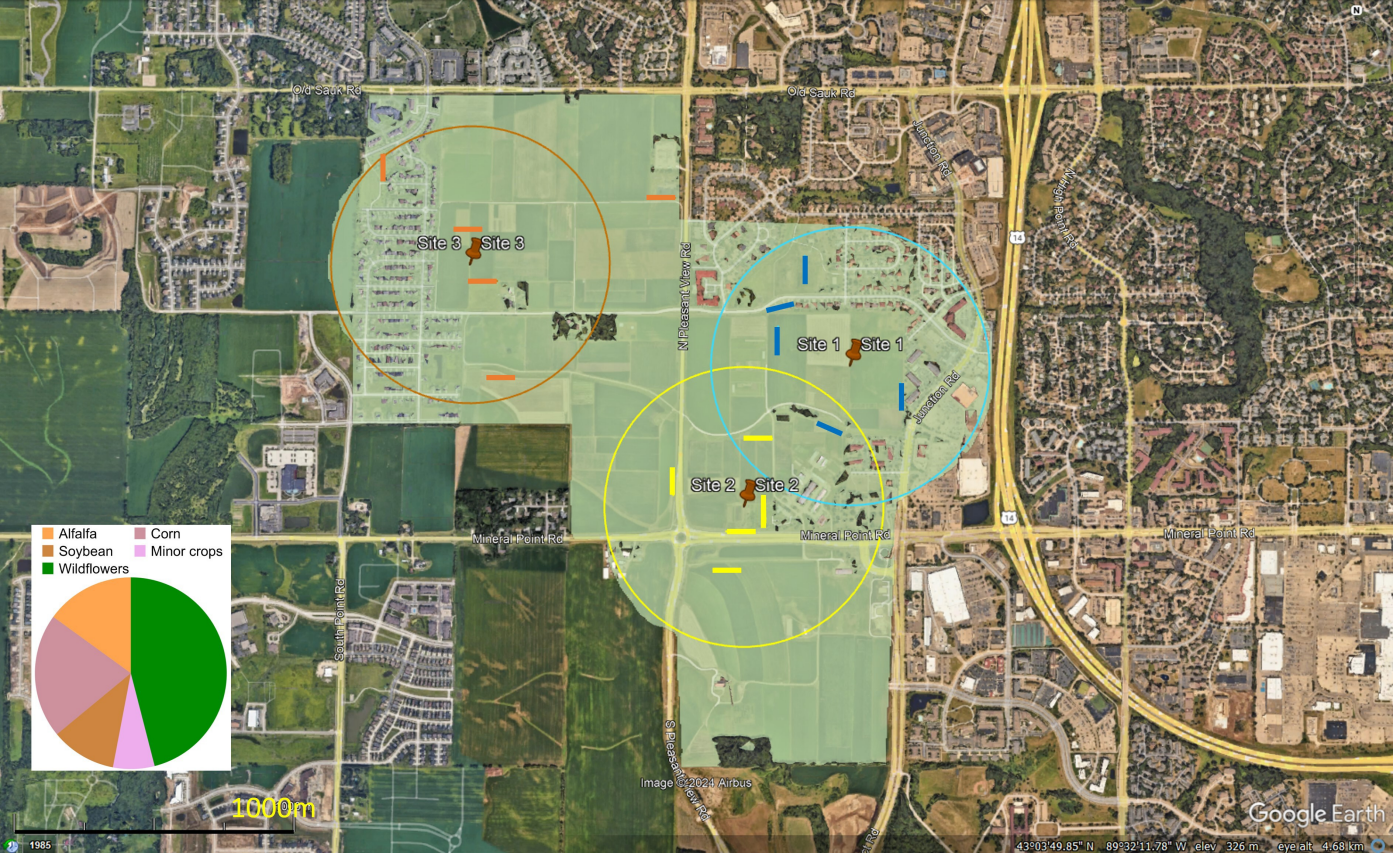
Birdseye view of the landscape and total area for which resource availability was estimated (green overlay). The total area of 339 hectares includes all habitat within the 500 m radius (three coloured circles) around the central point of each site (pin icon) and any crop fields that are within the boundaries of the research station. The pie chart depicts the relative abundance of wildflowers and individual crop categories as a proportion of the total resource availability area. The coloured lines indicate the location of wildflower survey transects, and the 1 km scale bar is on the bottom left.

This study was conducted from June to September 2016 at the West Madison Agricultural Research Station in Madison, WI, USA comprising a north temperate suburban agricultural landscape. We selected three sites at the research station for which to pair a single honeybee hive to a single bumble bee hive, for a total of six hives among the three sites (figure 1). The paired hives were placed along alfalfa crop fields. At sites 1 and 3, the honeybee and bumble bee hives were spaced 100 m apart, while at site 2, they were separated by 60 m. The distance among sites, measured between the centre point of the two hives within a site to the equivalent point at the other two sites ranged from 700 to 1500 m (sites 1−2: 700 m; sites 1−3: 1500 m and sites 2−3: 1400 m). The research station plus any extra area included within each 500 m radius surrounding the paired hives consisted of 339 hectares of crop fields and wildflowers (green shaded area in figure 1). The surrounding area comprised mainly semi-natural habitats and suburban development.

Honeybee colonies were established at each site 2 weeks prior to the start of the experiment to allow colony establishment and brood production. Each colony was housed in a two-frame observation hive beginning with ~2000 workers and a queen. Frames consisted of a mixture of open and closed brood, honey stores and bee bread. As protection from adverse weather, each hive was placed in a 1.2 m^3^ shelter with an exit tunnel providing access outside. Bumble bee colonies were placed in their respective site 3 days prior to starting the experiment, as they were purchased as pre-established colonies (Koppert Biological Systems, Howell, MI, USA). Each bumble bee colony consisted of approximately 75 workers at the beginning of the experiment and remained in the box which was provided. To protect the colony from weather and animals, each bumble bee colony was placed into a small three-walled wooden shelter located 0.5 m above the ground, with the fourth wall consisting of wire mesh with 1 cm^2^ holes.

### 2.2. Pollen collection

Pollen was systematically collected from returning foragers at each of the six hives over five discrete periods throughout the summer, beginning in June and continuing through September (P1: June 14–27, P2: July 1–13, P3: July 23–August 2, P4: August 8–25, P5: August 30–September 13). Within each pollen collection period, each of the six hives were examined three times across nine observation days, with the paired honeybee and bumble bee hives at a site assessed on the same days. Pollen collection was randomized with respect to repeated sampling throughout the summer by rotating among sites each day for a total of 45 sampling days.

Two observers visited the paired hives at a site to collect pollen from returning honeybee and bumble bee foragers. One observer would monitor the entrance to the honeybee colony, and the other the bumble bee colony, with observers switching each day. Monitoring began at 9 am (10 am in September) and continued until pollen pellets (corbicular pollen loads) were collected from 40 returning honeybee foragers and 20 returning bumble bee foragers, or until 4 pm, whichever came first. We chose to double the sample size for honeybees due their larger colony sizes relative to bumble bees. Upon collection, bees were placed individually into small vials and immobilized in a cooler with ice, whereby both pollen pellets were removed and placed individually into labelled 1.5 ml microcentrifuge tubes. To prevent resampling the same bee, captured bees were only released after all pollen pellets were collected for that day. Pollen pellets were stored at the lab inside a −20°C freezer until ready for drying and weighing. One pollen pellet per bee was dried at 45°C for 24 hours, the dry weight measured and subsequently identified using visual microscopy.

### 2.3. Pollen identification

To identify the floral resources from which bees collected pollen, the dried pellet from a bee was crushed in a 1.5 ml microcentrifuge tube, followed by the addition of Safarin-O pollen staining solution and vortex stirring to ensure pollen grains were randomly distributed throughout the solution. Immediately following preparation, 10 µl of homogenate was placed onto a microscope slide, topped with a coverslip and sealed along the edges with clear nail polish [31]. Using light microscopy, we counted 500 pollen grains in each sample at 400 x magnification, which were then categorized into morphotypes based on size, shape and ornamentation. After obtaining the number of pollen grains of each morphotype, we calculated their respective proportion within individual pollen pellets. Any morphotype not present in at least 3% of the sample was excluded from further analyses.

We assigned taxonomic identifications to each morphotype using reference slides of pollen collected directly from flowers at our study sites, together with online atlases (isaacslab.ent.msu.edu; paldat.org and globalpollenproject.org). All observed morphotypes could be identified to family, but when more than one morphotype was identified belonging to the same family, and neither could be identified to a genus or species, they were assigned a unique name that included the family and a distinguishing feature (electronic supplementary material, note S1). For example, Asteraceae_spines or Asteraceae_lophate would indicate a pollen morphotype that has either spine-like or lophate (ridging) ornamentation, respectively. Analyses at the morphotype level included all identified morphotypes, while for analyses at the family level, all morphotypes belonging to the same family were pooled.

### 2.4. Flower constancy and richness and diversity of bee-collected pollen

Following pollen identification, and after determining the composition of pollen pellets to morphotype and family levels, for each of the five periods, we calculated flower constancy which represents the proportion of bees from a colony that performed a flower constant pollen foraging trip on a given day. A foraging trip was identified by a bee returning to the hive with pollen pellets, whereby a flower constant foraging trip occurred when at least 97% of a pollen pellet consisted of a single morphotype or family. In addition, for each of these five periods, we quantified the richness and diversity of pollen collected over all bees sampled from a colony on a given day. Pollen richness (*S*) was calculated as the total number of morphotypes or families identified. Pollen diversity considers both the richness and relative abundance of each morphotype or family and was calculated using the ‘vegan’ package [32] in R [33]. We selected the Simpson diversity index (SDI = 1 D), whose value is constrained between 0 and 1, with values closer to one indicating higher diversity. SDI increases when the number of morphotypes or families increases, while the magnitude of the change depends on the relative abundance of each morphotype or family [34].

### 2.5. Plant resources

We considered both wildflower and crop floral abundance over the 339 hectare area to estimate the resources available to bees. This area encompassed the three sites of this study plus all land at the research station (figure 1). For each of the three major crops grown at the research station, we obtained data on the proportion of the 339 hectares occupied by each crop and on their floral abundance. We also determined the proportion of the research station occupied by minor crops and wildflowers. In addition to total resources, we also examined the floral abundance of only wildflowers, calculating plant richness and plant diversity based on these data, and compared these variables among sites and surveys, as described below.

#### 2.5.1. Crop floral abundance

The main crops growing at the research station were alfalfa (*Medicago sativa*), corn (*Zea mays*), and soybean (*Glycine max*), which comprised 89% of all crop hectares, with the remaining 11% comprising 10 minor crops (figure 1, electronic supplementary material, table S1). Alfalfa is a perennial plant grown for hay in Wisconsin, where fields are maintained for approximately 3 years. Seed production in alfalfa relies entirely on bees [35]. Hay is cut when about 10% of the field is in flower, and there are approximately three harvests over the summer months. To determine floral abundance, alfalfa fields were sampled by haphazardly dispersing a single 1 m^2^ quadrat five times in each of three fields (15 quadrats total) and counting the number of racemes within the quadrat to estimate the density of racemes in the field. To haphazardly disperse the quadrat, it was tossed in uncoordinated directions, with each subsequent toss originating from the location it had landed previously.

Corn and soybean fields are planted annually and have one mass flowering period in late July to early August. Corn is wind-pollinated and pollinators do not impact its yield. Soybean has a mixed mating system with cleistogamous and chasmogamous flowers, and the presence of pollinators can increase seed yield, although the increment varies among cultivars [36]. For both of these crops, we sampled floral abundance along a 100 m transect in each of three fields during early August. Each transect began at the field edge and was aligned in a north to south direction towards the field centre. Three 1 m^2^ quadrats for soybean, and three 1 2 m^2^ quadrats for corn were then placed along each transect at 0, 50 and 100 m from the field edge, resulting in a total of nine quadrats among the three fields for each crop.

Within quadrats in the soybean field, we counted the total number of stems and determined the number of racemes per stem on 50% of these stems. The total number of racemes per quadrat was obtained by multiplying the average number of racemes per stem by the number of stems per quadrat. The larger size of quadrats in the corn fields was due to the large size of corn stalks, with the 1 m width spanning two planted rows of corn and the 2 m length extending along the planted rows. For corn, we counted the number of stalks per quadrat and divided it by two prior to analysis to match the 1 m^2^ quadrat size in alfalfa and soybean.

#### 2.5.2. Wildflower floral abundance

Wildflowers were surveyed over 1−2 days during each of four times between June and September, or once every 3−4 weeks. At each of the three sites, we established five 100 × 1 m transects within 500 m of the hive pair, resulting in a total of 15 transects across sites. Eight of these transects were placed along crop plot edges, with the remaining seven located in fallow fields or vacant lots within or immediately adjacent to a site. During a survey, we placed 10 1 m^2^ quadrats along the length of each transect, and within each quadrat recorded the identity of flowering plants, the number of open flowers, or if having clustered flowers, the number of racemes (e.g. Fabaceae), umbels (e.g. Apiaceae) or heads (e.g. Asteraceae). While the location of a transect was maintained throughout the experiment and visited during each survey, the quadrats within a transect were removed and replaced haphazardly at each sampling, but always spanned the length of the transect.

#### 2.5.3. Wildflower richness and diversity

Wildflower richness and diversity were calculated at the level of plant species, plant family and pollen morphotype, based on the floral abundance data obtained during a plant survey. For the morphotype analyses, plant species were grouped according to their pollen morphology to align with the pollen morphotypes observed in the bee-collected pollen. Data were pooled across transects at a site for each survey, resulting in 12 values for plant richness or plant diversity (3 sites × 4 surveys). Wildflower richness and diversity followed the same method described earlier for bee-collected pollen (§2.4).

### 2.6. Proportion of pollen morphotypes in bee-collected pollen and resources

The proportion of a pollen morphotype available in bee-collected pollen was calculated separately for each bee species. Because pollen pellets were collected over five periods, but plant resources were quantified over four surveys, to compare bee-collected pollen to resources, we pooled both of the July periods, P2 and P3, of bee-collected pollen to match the July resource survey. The proportional abundance of a pollen morphotype in bee-collected pollen was calculated over all sites and days, and separately for each of the four surveys, by dividing the number of pollen grains of a given morphotype over the total pollen grains across all morphotypes.

For resources, the proportion of a pollen morphotype was calculated using the crop and wildflower floral abundance data (§ 2.5.1 and 2.5.2). We first determined the proportional area of the 339 hectares allocated to each of the three crops and to wildflowers. The proportional area covered by a crop was calculated by dividing the total area covered by the respective crop by the 339 hectares surveyed area (figure 1, area of green shading). To obtain the proportional acreage allocated to wildflowers, we subtracted the area devoted to crop fields (all crops) from the total 339 hectares area and divided that number by 339 hectares. The proportional abundance of each morphotype was then calculated for the respective survey as follows. For wildflowers, floral counts in a survey were obtained over 150 1 m^2^ quadrats (§ 2.5.2). We obtained floral counts for each of the plant species flowering during the respective survey. For each crop flowering during the survey, we calculated crop floral counts over 150 1 m^2^ quadrats. We then multiplied these floral counts by the proportional area occupied by the crop or wildflowers at the farm. We call these values estimated floral counts (efc). To obtain the proportion of a morphotype, we added the efcs of the different plant species (crop and/or wild) that are grouped into a morphotype and divided by the total floral counts, i.e. the efcs of all wild and crop plant species flowering during the survey.

### 2.7. Statistical analysis

#### 2.7.1. Flower constancy, richness and diversity of bee-collected pollen

Using linear mixed-effects models, we tested the effects of bee species, period, site and their two-way interactions on each of three response variables: (i) flower constancy, (ii) richness of bee-collected pollen (S), and (iii) diversity of bee-collected diversity (SDI), with separate analyses performed at the pollen morphotype and family levels. The response variables were measured on different non-consecutive days. In the model, bee species, period, site and their two-way interactions were fixed factors while day was a random factor. Day within each combination of site/period/species was considered a subplot sample, and the random three-way interaction between species, period and site was the ‘whole plot’ error term used to test each of the fixed factors. Models were constructed and tested using ‘proc mixed’ in SAS (SAS v. 9.4). We visually assessed the normality of model residuals with qqplots, and no transformations were needed. Tukey’s corrected multiple means comparisons were performed when either period, site or their interactions with species were statistically significant.

#### 2.7.2. Wildflower richness and diversity and composition among sites

To compare wildflower richness and diversity among sites and surveys, we performed six separate two-way ANOVA tests specifying richness or diversity at the species, family or morphotype level as the response variable, with site and survey as fixed factors for all tests. The normality of model residuals was visually assessed using qqplots, and no transformations were needed.

We also tested whether the relative representation of plant species, plant families or morphotypes in wildflower resources differed between the three sites by performing a multivariate per MANOVA test [32], separately for each category. For these tests, site was the explanatory variable and the proportional abundance of each of the species, family or morphotype the response variable.

#### 2.7.3. Comparing morphotype composition of bee-collected pollen between bee species

We used a contingency χ^2^-test to compare, for each of the four surveys, the proportion of each morphotype present in honeybee- relative to bumble bee-collected pollen. A statistically significant result would indicate that the relative abundance of at least some of the pollen morphotypes collected within each survey differed between the two bee species.

#### 2.7.4. Floral choices of each bee species

We used goodness of fit χ^2^-tests to compare the proportional abundance of each morphotype in honeybee or bumble bee-collected pollen to its prevalence in the resources. The proportion of each morphotype observed in the resources represented the expected proportions (the null hypothesis). A preference for a pollen type is indicated by a bee species collecting a morphotype in greater proportion than its representation in the resources and thus can only be determined for the pollen morphotypes present in both resources and bee-collected pollen. The potential preferences of each bee species were examined separately at each of the four surveys. To obtain counts for these tests, each proportion for bee-collected pollen morphotypes or resources was multiplied by 500. A statistically significant result would indicate how bees did not collect pollen morphotypes based on their representation in the resources overall but preferred or avoided some pollen types.

Besides using goodness of fit χ^2^-tests, we also analysed the data using an ecological network analysis approach using the ‘econullnetr’ package in R [37]. The test was run using pollen pellet (individual bee) as a replicate, thus the proportion of each morphotype in a pollen pellet. The null hypothesis is generated from the data assuming that bees collect pollen morphotypes based on their availability in the resources. A proportion of bee-collected pollen greater or less than the 95% confidence interval around the proportion of that morphotype expected to be collected based on resource abundance would respectively indicate preference or avoidance for that pollen morphotype.

For all the χ^2^-tests, the pollen morphotypes that were observed in both categories being tested and had pollen counts greater than 5, to satisfy the χ^2^-test requirement, were included. In addition, to account for morphotypes present in both categories being tested but with counts lower than 5 in at least one category, and morphotypes that were observed in only one of the categories being compared, we added a morphotype row called ‘other’. For all studies of bee preferences based on pollen collection, even when resources are intensively surveyed, there will be instances where a pollen type unidentified in the resources was collected by a bee species. In our analysis, these are added to the ‘other’ entry for the bee-collected pollen. In addition, cases where pollen counts of morphotypes are only present in resources illustrate avoidance, or some other lack of need, and these would be added to the ‘other’ entry for the resources category. This approach keeps the original proportions observed for the pollen morphotypes present in both the bee-collected pollen and in the resources. Eliminating the pollen counts of some of the unvisited resources or of uncollected pollen, as is commonly done in preference tests [38], biases the proportions of morphotypes collected or resources available because the total frequency must equal 1.0 for both the bee-collected pollen and the resources categories (see an example in electronic supplementary material, note S2).

## 3. Results

### 3.1. Plant families in bee-collected pollen

Over the five periods of pollen collection, we collected 1048 pollen pellets from honeybees over 33 days and 486 pollen pellets from bumble bees over 34 days (electronic supplementary material, table S2). We observed 16 plant families over both honeybee and bumble bee-collected pollen throughout the experiment (electronic supplementary material, table S3). Plants in the Fabaceae family contributed 62% of all pollen collected by honeybees over their foraging season and was the pollen collected in the greatest proportion in all periods except period 5, when honeybees collected Asteraceae pollen the most (electronic supplementary material, figure S1). Honeybees also collected Brassicaceae pollen later in the summer, with the highest proportion (24%) collected in period 4 (electronic supplementary material, figure S1). These trends differed in bumble bees which overall collected 38% of their pollen from Fabaceae (range among periods: 28%–43%) and 34% from Brassicaceae (range among periods: 13%–44%). Except for period 3, where bumble bees collected the greatest proportion of pollen from the Asteraceae family, pollen from the Fabaceae and Brassicaceae dominated the bumble bee pollen collection throughout their foraging season (electronic supplementary material, figure S1).

### 3.2. Flower constancy

**Figure 2.**
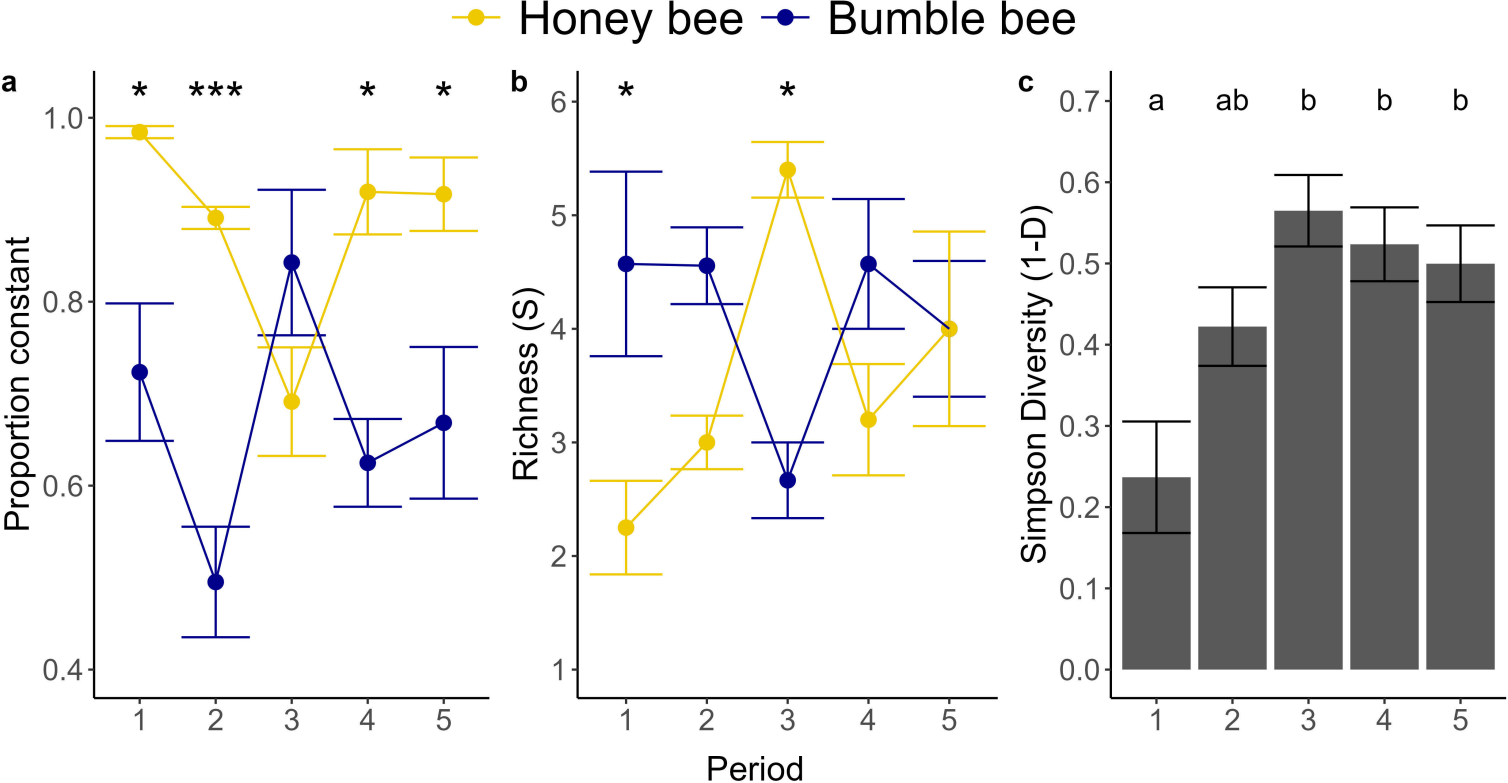
Family level (mean ± SE) daily estimates for (a) flower constancy between the two bee species over their foraging season; (b) pollen richness between the two bee species over their foraging season and (c) pollen diversity over the foraging season (both bee species combined). Distinct letters indicate significant differences based on Tukey’s multiple comparison tests. *p < 0.05; ***p < 0.001.

A greater proportion of honeybees than bumble bees were flower constant at the pollen morphotype (mean ± SE) (honeybee: 0.87 ± 0.02; bumble bee: 0.62 ± 0.04) and plant family levels (honeybee: 0.89 ± 0.02; bumble bee: 0.64 ± 0.04) (table 1a,b). There was, however, a statistically significant bee species × period interaction for the proportion of flower constant pollen foragers at both the morphotype (table 1a) and family (table 1b) levels. Honeybees were more flower constant than bumble bees in all periods except period 3 where the reverse trend was observed (figure 2*a*). There was no effect of site or its interaction with period or species, on flower constancy (table 1a,b).

**Table 1.** Effects of bee species, site, period and their two-way interactions on flower constancy, pollen richness and pollen diversity of bee-collected pollen at (a) the morphotype and (b) the family levels. The effects of site and survey on the (c) richness and (d) diversity of plant species, plant families and their assigned pollen morphotypes (morphotype) in the wildflower resources. Significant effects of the linear mixed models are indicated in bold. There were five periods for bee-collected pollen and four surveys for resources as detailed in the text.

(a) bee-collected pollen: pollen morphotypes									
	constancy			richness			diversity		
factor	DF	F	P	DF	F	P	DF	F	P
site	2.7	0.56	0.593	2.7	0.26	0.779	2.7	0.17	0.849
period	4.7	5.06	**0.031**	4.7	0.81	0.556	4.7	2.60	0.128
species	1.7	20.41	**0.003**	1.7	0.78	0.405	1.7	3.27	0.114
period x species	4.7	5.37	**0.027**	4.7	3.71	0.063	4.,7	0.52	0.727
site x species	2.7	1.83	0.230	2.7	0.81	0.482	2.7	0.55	0.598
site x period	8.7	1.39	0.338	8.7	1.13	0.444	8.7	0.57	0.779

(b) bee-collected pollen: plant families									
site	2.7	0.29	0.756	2,7	0.13	0.876	2.7	0.19	0.831
period	4.7	2.95	0.101	4.7	0.44	0.778	4.7	4.70	**0.037**
species	1.7	24.19	**0.001**	1.7	1.35	0.283	1.7	11.1	**0.013**
period x species	4.7	5.34	**0.027**	4.7	4.11	**0.050**	4.7	2.00	0.199
site x species	2.7	1.85	0.227	2.7	0.66	0.544	2.7	0.13	0.881
site x period	8.7	1.15	0.433	8.7	0.81	0.613	8.7	0.69	0.695

(c) wildflower surveys: richness									
	species			family			morphotype		
site	2.5	2.22	0.20	2.5	3.26	0.12	2.5	5.28	0.06
survey	3.5	0.63	0.63	3.5	0.72	0.58	3.5	2.11	0.22

(d) wildflower surveys: diversity									
site	2.5	0.22	0.81	2,5	0.51	0.63	2.5	0.19	0.83
survey	3.5	0.61	0.64	3,5	0.68	0.60	3,.5	0.70	0.59

### 3.3. Richness and diversity of bee-collected pollen

At the pollen morphotype level, pollen richness did not significantly differ between the two bee species even though bumble bees collected pollen from 5.6 ± 0.36 morphotypes relative to 3.9 ± 0.36 for honeybees (table 1a). At the plant family level, however, there was a statistically significant bee species × period interaction (*p* = 0.05) in the richness of bee-collected pollen (table 1b). Compared to bumble bees, honeybees collected pollen from fewer plant families during period 1, but from more plant families during period 3 (figure 2*b*). There were no statistically significant differences in the number of plant families the two bee species collected pollen from in periods two, four or five (*p* > 0.05 for all comparisons).

For diversity at the pollen morphotype level, we did not observe a statistically significant effect of bee species, period, site or their two-way interactions (table 1a). There were, however, significant main effects of both bee species and period on pollen diversity at the plant family level (table 1b). Bumble bees collected a greater family level diversity of pollen than honeybees (bumble bee SDI: 0.54 ± 0.03; honeybee SDI: 0.33 ± 0.04: table 1b) and both bee species collected the lowest family level diversity of pollen during period 1 (figure 2*c*). No other factors affected pollen diversity at the family level (table 1b).

### 3.4. Wildflower richness and diversity, and composition among sites

**Figure 3.**
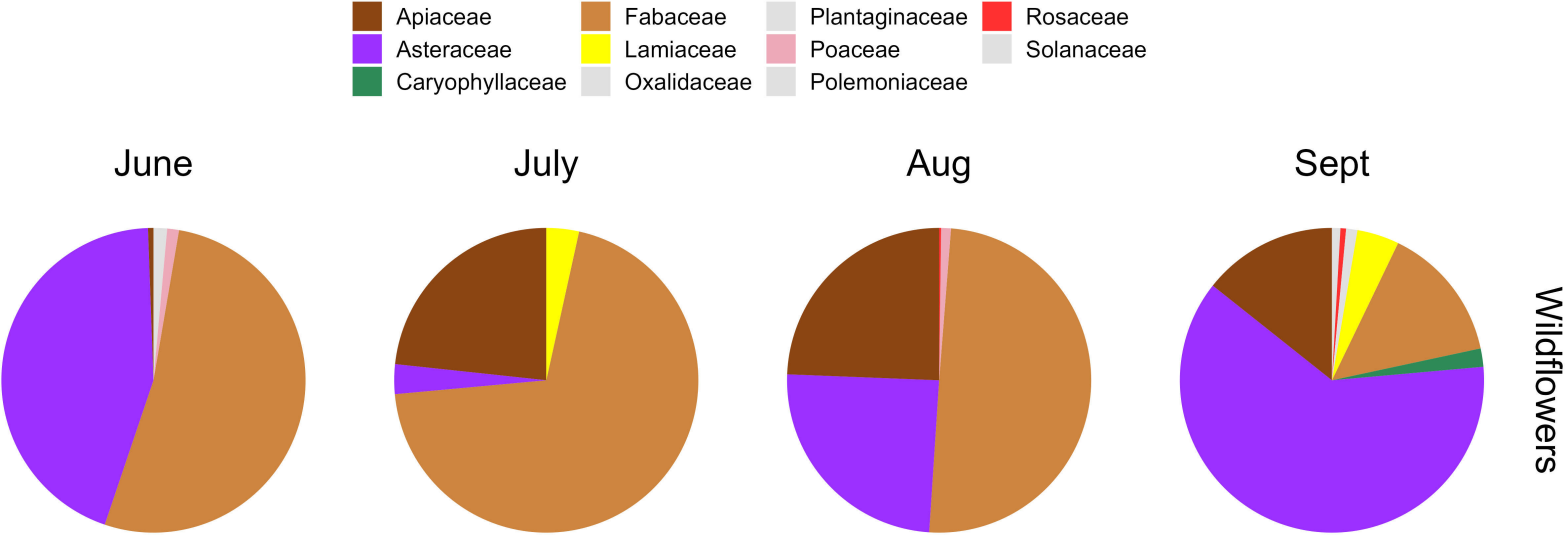
The plant family level composition of wildflowers (proportions) during each of the four resource surveys in the 339 hectare surveyed area.

There were no statistically significant differences in wildflower richness or diversity at any classification levels between the four surveys or among the three sites (table 1c,d). Nor was there a significant difference between sites in the composition of species, families or morphotypes of wildflowers (*p* > 0.3 for all comparisons; electronic supplementary material, figure S2). A total of 11 plant families were observed in wildflowers (figure 3), eight of which were also identified in bee-collected pollen. The three most common families in the resources were Fabaceae, Asteraceae and Apiaceae although their relative abundance varied over the season (figure 3). For example, Fabaceae was most common (50% +) in June, July and August, with Asteraceae being most abundant (62%) in September although they were also common in June (figure 3). Apiaceae were uncommon in June (<1%) but comprised closer to 25% of available wildflowers from July to September (figure 3). The abundance of the remaining nine plant families were each less than 5% throughout the study (figure 3).

### 3.5. Pollen morphotypes in bee-collected pollen and resources

**Figure 4.**
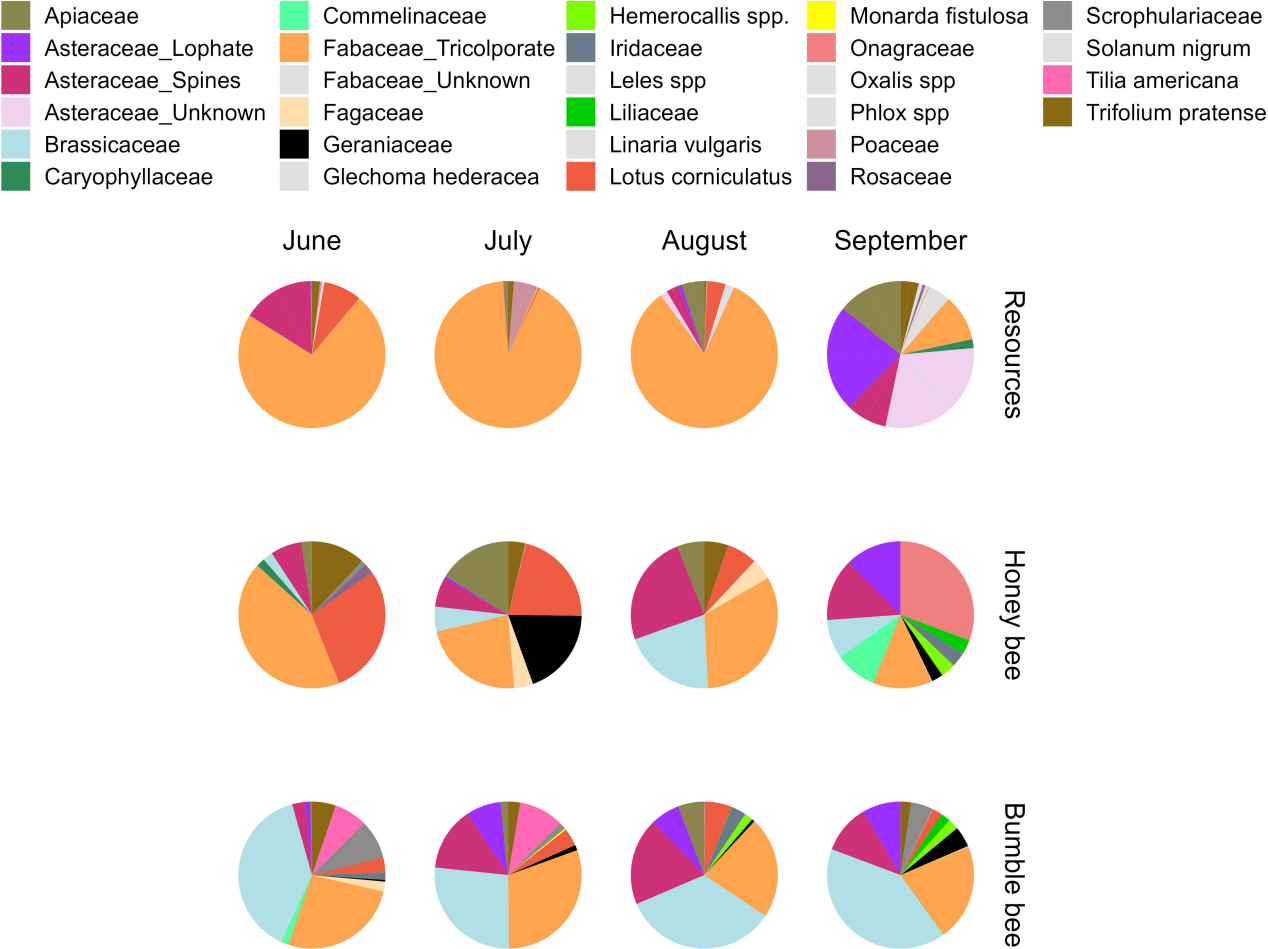
The proportion of morphotypes observed in resources (top row), and pollen collected by honeybees (middle row) and bumble bees (bottom row) during each of the respective surveys. The pollen grain counts (out of 500) for each morphotype are presented in electronic supplementary material, table S6.

Among all bee-collected pollen and resources (focal crops and wildflowers), we identified 28 total morphotypes (figure 4), 10 of which were observed in both pollen and resources, 10 only in pollen and 8 only in resources (table 2). For the resources, the wildflowers associated with each morphotype are summarized in the electronic supplementary material, table S3. When crop pollen is considered, the Fabaceae_tricolporate morphotype included pollen from *Medicago sativa* (alfalfa), *Glycine max* (soybean), *Securigera varia* (crown vetch) and *Trifolium repens* (white clover), while pollen of *Trifolium pratense* (red clover) and *Lotus corniculatus* (birdsfoot trefoil), also in the Fabaceae family, could be identified separately. The Asteraceae_lophate morphotype included three distinct plant species and the Asteaceae_Spines comprised six different plant species (electronic supplementary material, table S3).

**Table 2.** The morphotypes identified in both pollen and resources, pollen only and resource only. Resource only morphotypes are those plant taxa that were identified in wildflower surveys but were not found in bee-collected pollen. Morphotypes with (hb) or (bb) indicate morphotypes that were collected only by honeybees or bumble bees, respectively.

pollen and resources	pollen only	resource only
Apiaceae	Brassicaceae	Asteraceae_Unknown
Asteraceae_Lophate	Commelinaceae	Fabaceae_Unknown
Asteraceae_Spines	Fagaceae	*Glechoma hederacea*
Caryophyllaceae (hb)	Geraniaceae	Leles spp.
Fabaceae_Tricolporate	Hemerocallis spp.	*Linaria vulgaris*
*Lotus corniculatus*	Iridaceae	Oxalis spp.
*Monarda fistulosa* (bb)	Liliaceae	Phlox spp.
Poaceae	Onagraceae (hb)	*Solanum nigrum*
Rosaceae (hb)	Scrophulariaceae	
*Trifolium pratense* (Fabaceae)	*Tilia americana*	

#### 3.5.1. Comparing morphotype composition of bee-collected pollen between bee species

The amount of pollen collected from the distinct pollen morphotypes differed significantly between honeybees and bumble bees at each survey (June: *χ*2 = 352.2, *df* = 6, *p* < 0.0001; July: *χ*2 = 334.3, *df* = 7, *p* < 0.0001; August: *χ*2 = 31.6, *df* = 5, *p* < 0.0001; and September: *χ*2 = 229.2; *df* = 7; *p* < 0.0001) (electronic supplementary material, table S4). Both bee species collected a significant proportion of their pollen from Fabaceae_tricolporate throughout the flowering season (figure 4; electronic supplementary material, table S4). The other morphotypes most visited in a given month generally differed between the two bee species (electronic supplementary material, table S5). For example, in June, honeybees collected second most from *Lotus corniculatus* (29%), while bumble bees collected most from Brassicaeae (39%) (electronic supplementary material, table S4). In July, honeybees collected significant pollen from *Lotus corniculatus* and from Geraniaceae and Apiaceae while bumble bees collected second most from Brassicacee and then Asteraceae_spines (electronic supplementary material, table S4). Only in August did both bee species collect most of their pollen from the same three morphotypes, Fabaceae_tricolporate, Asteraceae_spines and Brassicaceae, although in different proportions (electronic supplementary material, table S5). And in September, honeybees gathered the largest proportion of their pollen from Onagraceae, a family that had not been collected by honeybees in previous surveys, and that was not detected in the resources (electronic supplementary material, table S5). Bumble bees kept gathering most of their pollen from Brassicaceae in that month, and the second most from Fabaceae_tricolporate.

#### 3.5.2. Floral choices of each bee species

**Figure 5.**
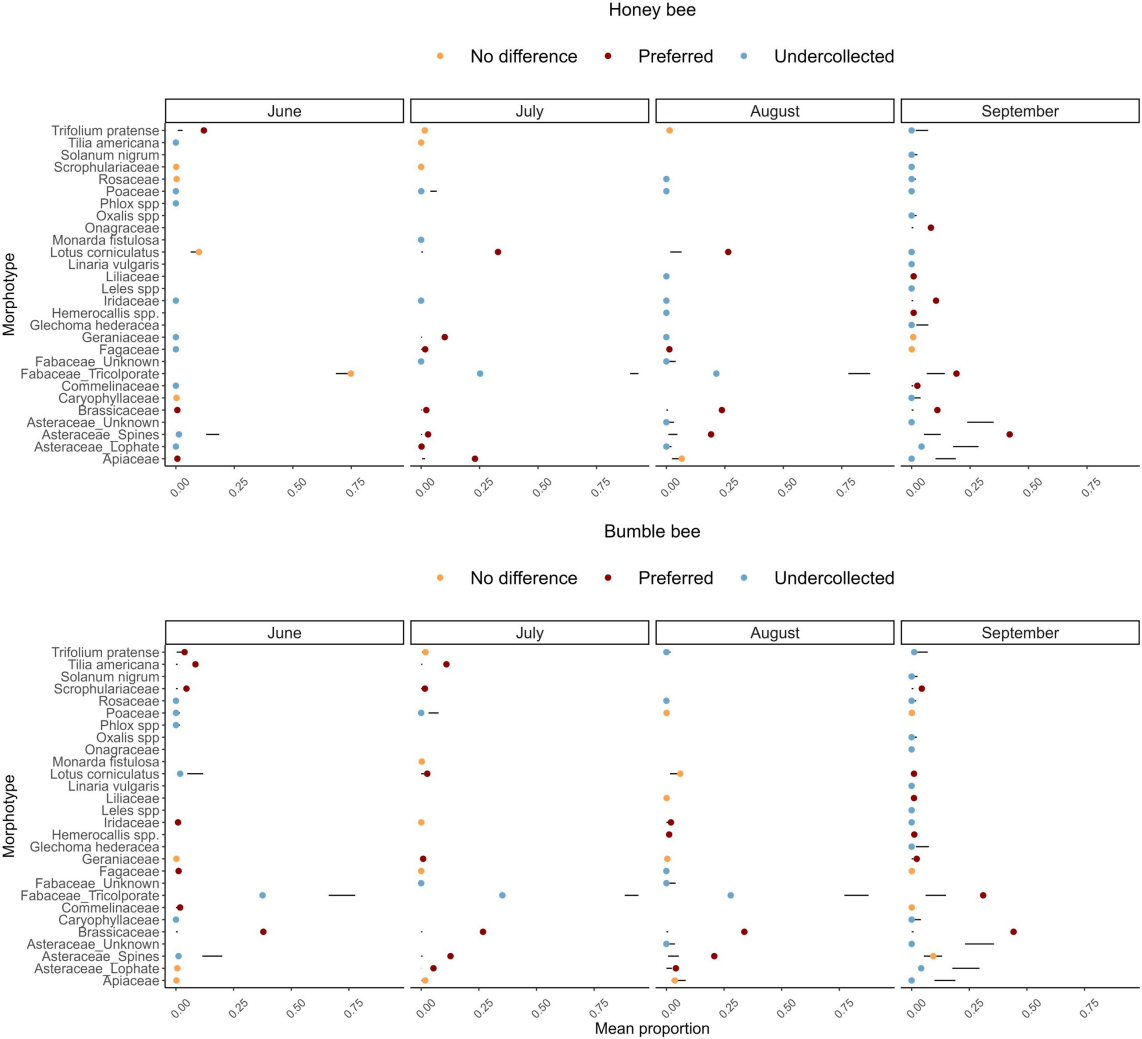
Results of the ecological network analysis using the ‘econullnetr’ package in R [37] tested at the level of the pollen pellets (individual bee) for each bee species and survey. Black lines represent 95% confidence intervals around the predicted proportion of each pollen morphotype in bee-collected pollen based on its availability in resources. Coloured dots show the observed proportion of a morphotype collected. A preference indicates a morphotype was collected in greater proportion than expected based on its availability in resources.

The goodness of fit χ^2^-tests indicated that neither honeybees nor bumble bees collected pollen based on its distribution in the resources, and this was true throughout the foraging season. Overall, the proportion of the different morphotypes in the bee-collected pollen differed from its abundance in the resources at each of the four surveys for both honeybees (June: *χ*2 = 876; *df* = 4; *p* < 0.0001; July: *χ*2 = 3457; *df* = 3; *p* < 0.0001; August: *χ*2 = 1644; *df* = 4; *p* < 0.0001; September: *χ*2 = 41.5; *df* = 3; *p* < 0.0001) and bumble bees (June: *χ*2 = 13417; *df* = 4; *p* < 0.0001; July: *χ*2 = 2785; *df* = 3; *p* < 0.0001; August: *χ*2 = 2444; *df* = 4; *p* < 0.0001; September: *χ*2 = 114; *df* = 4; *p* < 0.0001) (figure 4; electronic supplementary material, table S6). Results of the ecological network analysis indicated that in June, honeybees preferentially collected *Trifolium pratense* pollen but collected *Lotus corniculatus* and Fabaceae_tricolporate pollen based on their availability in the resources (figure 5). Most of the pollen honeybees collected in June came from these last two morphotypes (figure 4). In contrast, bumble bees frequently collected most of their pollen from Brassicaceae and Fabaceae_tricolporate in June (figure 4) and gathered less pollen from Fabaceae_tricolporate relative to its representation in the resources (figure 5). Because Brassicaceae pollen was not detected in the resources at any survey (electronic supplementary material, table S5), we assumed the plants were outside the survey area; we therefore cannot assign a preference to that morphotype as we do not know its actual representation in the resources. In July, honeybees collected most of their pollen from Fabaceae_tricolporate, *Lotus corniculatus* and Geraniaceae and indicated a preference for *Lotus corniculatus* and Apiaceae and we assumed that Geraniaceae originated from outside the survey area because it was never detected in the resource surveys. Bumble bees, in contrast, collected most of their pollen from Fabaceae_tricoloporate, Brassicaceae and Asteraceae_spines (figure 4) and indicated a preference for Asteraceae_spines in that survey (figure 5). The types of pollen collected by each bee species were most similar in August (figure 4), yet their preferences still varied (figure 5). Overall, the preference for a given morphotype often changed over the foraging season for a bee species and differed between bee species (figure 5).

## 4. Discussion

This study provides a detailed account of the pollen collection patterns of two bee species over their foraging season in one agricultural landscape. We emphasize how knowledge gained from this approach can be used to optimize the composition of AESs such as flower strips, especially when the goals are to sustain specific crop pollinators and increase crop yield. Bee-collected pollen better illustrates pollen collection patterns relative to pollinator visitation because the amount of pollen collected per flower can vary between plant species [39] and for a given pollinator type [40]. We compare the information obtained here with other studies to highlight plant groups most visited or preferred by a bee species [26,27]. Although this study was done in one landscape, once commonly visited and preferred plant groups have been identified, local representatives of these groups can be used in the flower strips or other AES when extrapolating to other landscapes. We first describe similarities and differences in the pollen foraging strategies of these two bee species before applying such information to optimize the composition of AES such as flower strips.

### 4.1. Pollen foraging activity

The western honeybee and common eastern bumble bee foraging in a suburban-agricultural landscape have clear species level differences in pollen foraging pattern throughout their foraging season. In line with our prediction for flower constancy, honeybees were overall more flower constant than bumble bees regardless of testing at the family or morphotype levels. Honeybees tended to gather pollen from a single morphotype during a foraging trip, which reflects their recruitment behaviour such as the waggle dance [41] and supports the results of a previous study [42]. The lesser constancy of bumble bees led to them collecting a greater diversity of pollen, at least when pollen morphotypes were examined at the family level. The most likely explanation for the discrepancy between having a significant impact of species at the family but not morphotype levels would be the presence of fewer categories with more samples per category at the family level, providing more power for the statistical test. Moreover, the fact that some families combined more morphotypes than others may have influenced the results by increasing the abundance of pollen collected from a single family by a bee species. For example, honeybees collected a greater quantity of *Lotus corniculatus* and *Trifolium pratense* than bumble bees which together with Fabaceae_tricolporate combined into the family Fabaceae and could have lowered the types of pollen collected by honeybees at the family level, thus increasing the difference between the two species. In contrast to the current study, Graham *et al*. [43] observed bumble bees collecting from a greater number of morphotypes relative to honeybees when brought as managed pollinators to blueberry fields, even though their morphotype categories included a mixture of plant species, genera and family, similar to this study. Thus, distinctions exist between studies as to whether species differences can be detected at the morphotype or only the family level comparisons.

In addition to exhibiting differences in flower constancy and pollen diversity, the two bee species varied in the proportion of pollen they collected from distinct morphotypes over the flowering season (figure 4). Neither bee species collected all of its pollen solely based on the abundance of the different morphotypes over the landscape, and this was true throughout their foraging season; they avoided some pollen types, preferred others and collected in proportion with resource abundance for yet other morphotypes. Both honeybees and bumble bees chose between plant species and preferentially visited some plant species over others [44,45]. One surprising finding from this study was that the two bee species collected pollen from similar resources over the entire foraging season, although the most collected pollen types varied between species at any given survey. Only in August did the two bee species collect most of their pollen from the same three morphotypes, but in different proportions. We discuss in the next section how information about the most collected and preferred morphotypes collected by each bee species over their foraging season can guide the composition of flower strips or other AES aimed at maintaining specific pollinators and increasing crop yield.

### 4.2. Pollinator foraging strategies and agri-environment schemes

The morphotypes most collected by one or both bee species at some point in the season were Fabaceae_tricolporate, Brassicaceae, *Lotus corniculatus*, Asteraceae_spines and Onagraceae pollen. Fabaceae and Brassicaceae provide pollen of high nutritional value for bees [46,47], and a monofloral Brassicaceae diet can have positive impacts on honeybee health equivalent to a mixed pollen diet [47]. Numerous Asteraceae wildflower species have been shown to control levels of infection with common intestinal parasites of bumble bees [48]. Moreover, plants in the Fabaceae, Asteraceae and Brassicaceae are collected by other agriculturally important social bees, such as stingless bees (Melittidae) [49]. These morphotypes provide valuable benefits to bees and are thus beneficial additions to flower strips or other AES aimed at sustaining specific bee species and improving crop yield.

The species comprising the Fabaceae_tricolporate morphotype in this study included *Medicago sativa* (alfalfa), *Glycine max* (soybean), *Securigera varia* (crown vetch) and *Trifolium repens* (white clover). Fabaceae plants as a group, notably *Lotus corniculatus*, *Medicago sativa* and *Trifolium repens*, in conjunction with *Brassica* have been shown to support honeybees [27]. *Lotus corniculatus*, which can persist over years in managed wildflower plots [22,50], and was collected by both bee species in this study, has been previously identified as an important resource for both bumble bees [51,52] and honeybees [53,54] in developed landscapes. A large portion of the pollen collected by honeybees was of the Fabaceae_tricolporate morphotype, and we mostly observed honeybees foraging for nectar on *Medicago sativa*, while they gather both pollen and nectar from *Trifolium repens*. Bumble bees collect both nectar and pollen from these two plant species. These Fabaceae plants represent important resources to include in AESs meant to support honeybees and bumble bees and together they provide both nectar and pollen to bees.

Both bumble bees and honeybees visited only a fraction of the available resources, and while they collected pollen most abundantly for some of the available morphotypes, they also gathered pollen in smaller quantities from other morphotypes. Such a pattern has been previously reported for bumble bees, where they visited 25 out of the available 78 plant species but only made frequent visits to 9 species, and rare visits (<5) to 16 other species [55]. This pattern may be driven by bees actively balancing nutritional intake as pollen composition has been shown to influence the foraging pattern of bumble bees [56]. It would be interesting to understand differences in the nutritional quality of the pollen provided by the different morphotypes bees visited, to determine if the morphotypes visited infrequently, or the rare morphotypes for which the bees indicated a preference provided nutrients that balance against the pollen collected in greater abundance. The ‘nutritional landscape’ (reviewed in [57]) can influence patterns of collection of minor pollen resources and is an important consideration for determining the composition of AES to maintain pollinators.

While we only examined one landscape, the landscape context may influence the pollen collection patterns of bees and its impact may vary between bee species. Requier *et al.* [58] detected high pollen diet dissimilarity between honeybee apiaries, linked to the geographic distances between apiaries and the landscape composition dissimilarities, suggesting that the honeybee diet is strongly affected by the landscape context. In contrast, Bezerra da Silva Santos *et al*. [18] did not observe a correlation between the pollen types collected by honeybees at each hive and the distances between the hives, suggesting little influence of the landscape. For bumble bees, Saifuddin and Jah [52] found greater differences in pollen composition among sites than among bumble bee species suggesting an impact of the landscape context. Other agriculturally important bees like the solitary *Osmia cornuta* showed high fidelity to Rosaceous orchard trees regardless of the landscape context, in contrast to *Bombus terrestris* for which the diversity of collected pollen increased with the diversity of resources, and this trend was detected both at the local and landscape levels [59]. When the landscape is known to impact pollen composition for a bee species, we recommend that the specific plant species from a particular family or genus to include in the AES be a local representative of this morphotype.

Even though the three focal crops in our study area did not rely on pollinators, given alfalfa is used for hay production in the midwest region, corn is wind pollinated and soybean is primarily self-pollinated, the information obtained in this study helped identify frequently visited and preferred plants used by these two pollinators. While honeybees remain strictly a managed pollinator in the midwest USA, *Bombus impatiens* is the most common wild bumble bee species in the area. Our results indicate how both most collected and less frequently collected but preferred morphotypes should be included in an AES. The inclusion of less frequently collected pollen morphotypes/plant species in the AES ensures the availability of important and diverse nutrients to bees [57].

In future studies, we recommend that morphotypes present in low quantity in the resources but disproportionately collected by bees be analysed for amino acid and fatty acid composition, and their content compared to commonly collected pollen types. Furthermore, for morphotypes that include more than one plant species (as determined by the resource surveys), additional information should be gathered on the frequency of visits of the two bee species on these plant species and on whether pollen, nectar or both are collected by each bee species. Well-designed flower strips and other AES schemes that meet pollinator nutritional needs will help sustain pollinators of interest and maintain them in the vicinity of the crop with the goal of improving crop yield.

### 4.3. Abundant resources not collected by bees

The lack of corn pollen observed in our study was surprising, particularly for honeybees, as previous studies have observed a high abundance of corn pollen, a wind-pollinated crop, being returned to honeybee colonies during the bloom period [29,47,60]. However, exclusive consumption of corn pollen decreases the longevity of individual honeybees [61] and may negatively impact nurse honeybee physiology [47]. One hypothesis to explain the lack of bee-collected corn pollen, which was abundant over the landscape, is that bees avoid such nutritionally deficient resources when preferred resources are available. This is an important finding as previous studies suggest that corn pollen is a valuable resource for bees; the results of the current study indicate otherwise. The fact that corn is wind-pollinated and has been shown to be nutritionally deficient for bees further support the finding of this study. We propose that the abundance of corn pollen in bee-collected pollen be used as an indicator for the need of AES in an area to increase floral resource enrichment.

### 4.4. Limitations

There are inherent challenges in studies linking resource utilization by bees to availability over an area. First, identifying all the resources available to pollinators, especially those with longer foraging distances, may never be possible. The current study examined a 339 hectare area and yet we observed morphotypes in bee-collected pollen that were not identified in the resources. The absence of a morphotype in the resources could represent uncommon morphotypes in the resources or resources located beyond our surveyed area. Second, preference studies have limited the analyses to the comparison of only the morphotypes present in both bee-collected pollen and resources [52,62,63]. We believe all morphotypes present in the bee-collected pollen and resources should be included in the analyses; removing morphotypes from the bee-collected morphotypes or from the resources biases the proportion of the remaining bee-collected pollen and resource morphotypes, as was explained earlier. We proposed a solution to maintain the proportion of the bee-collected pollen and its representation in the resources to reflect the observed data, with the understanding that a preference cannot be ascertained for pollen morphotypes most likely to have originated from outside the area surveyed for resource availability.

## 5. Conclusion

The western honeybee and the common eastern bumble bee have distinct pollen foraging strategies. The honeybee species is more flower constant and collects less diverse pollen relative to the bumble bee species. Over the entire foraging season, both bee species collected similar resource types, but each species varied in their most collected resources at each survey. We highlighted morphotypes of interest and discussed the importance of incorporating both the abundant bee-collected morphotypes and morphotypes obtained in smaller proportions in flower strips and other AES aimed at sustaining pollinators and increasing crop yield. Unlike many other studies, corn pollen was not detected in bee-collected pollen despite its abundance in the resources. This pattern suggests that corn may only be visited when other preferred resources are not available and its abundance in bee-collected pollen could be used as an indicator for the need of AES in an area to increase floral resource enrichment. We discussed limitations of studies comparing bee-collected pollen to availability in resources and proposed a method that includes all morphotypes in the preference comparisons, instead of limiting analyses to only the morphotypes present in both bee-collected pollen and resources, as the latter distorts the observed morphotype proportions. We encourage similar studies in distinct landscapes and with different bee species to improve the composition of flower strips and other AES aimed at sustaining pollinators and increasing crop yield. Moreover, asking similar questions regarding resource use for flower visitors involved in pest control that would be seeking egg laying sites [64] or nectar sources [65] will be helpful in guiding the floral composition and structure of AES schemes aimed at increasing insect diversity.

## Data Availability

Data and code have been submitted to Dryad [66]. Supplementary material is available online [67].
